# Molecular cytogenetic characterization of partial trisomy of the long arm of chromosome 11 in a patient with multiple congenital anomalies

**DOI:** 10.1186/s13039-022-00595-0

**Published:** 2022-04-19

**Authors:** Austin Walker, Xianfu Wang, Young Mi Kim, Xianglan Lu, Ashley Taylor, Danielle Demarzo, Shibo Li, Hui Pang

**Affiliations:** 1 College of Medicine, University of Oklahoma Health Sciences Center, 941 Stanton L. Young Boulevard, Room 241, Oklahoma City, OK 73104 USA; 2 Genetics Laboratory, Oklahoma Children’s Hospital, OU Health, 1122 NE 13th Street, Suite 1400, Oklahoma City, OK 73104 USA; 3 Pediatric Specialties Clinic, Oklahoma Children’s Hospital, OU Health, 1200 N. Children’s Ave., Suite 5D, Oklahoma City, OK 73104 USA

**Keywords:** Trisomy 11q, Partial trisomy, 11q23-qter, Unbalanced translocation

## Abstract

**Background:**

Partial trisomy of the long arm of chromosome 11 is a rare cytogenetic abnormality. It has been characterized by variable sized duplications that lead to a range of phenotypes including growth retardation, developmental delay/intellectual disability, and distinctive craniofacial abnormalities. Congenital heart defects, skeletal abnormalities, urogenital anomalies, and hypotonia are found in some affected individuals.

**Methods:**

We describe a 16-year-old patient presented with most of the hallmark phenotypes of trisomy 11q syndrome as well as exhibiting symptoms of hearing loss, seizures, and abnormal endocrinological and ophthalmological findings. Routine chromosome analysis and subsequent chromosomal microarray analysis (CMA) were performed to detect genetic abnormalities in this patient.

**Results:**

We identified an abnormal male karyotype with a derivative chromosome 4 due to an unbalanced translocation between chromosomes 4 and chromosome 11. The CMA results revealed a 56 Mb duplication of chromosome 11q14.1-qter and a 874 Kb terminal deletion of the short arm of chromosome 4.

**Conclusion:**

A genomic imbalance resulting in partial trisomy 11q was found in a patient with multiple congenital anomalies. We compared the phenotypes of all known “pure” trisomy 11q cases in the literature and find that trisomy 11q23-qter is both recurrent and the most common cytogenetic abnormality found in the reported cases. It is associated with the core features of trisomy 11q syndrome.

**Graphical abstract:**

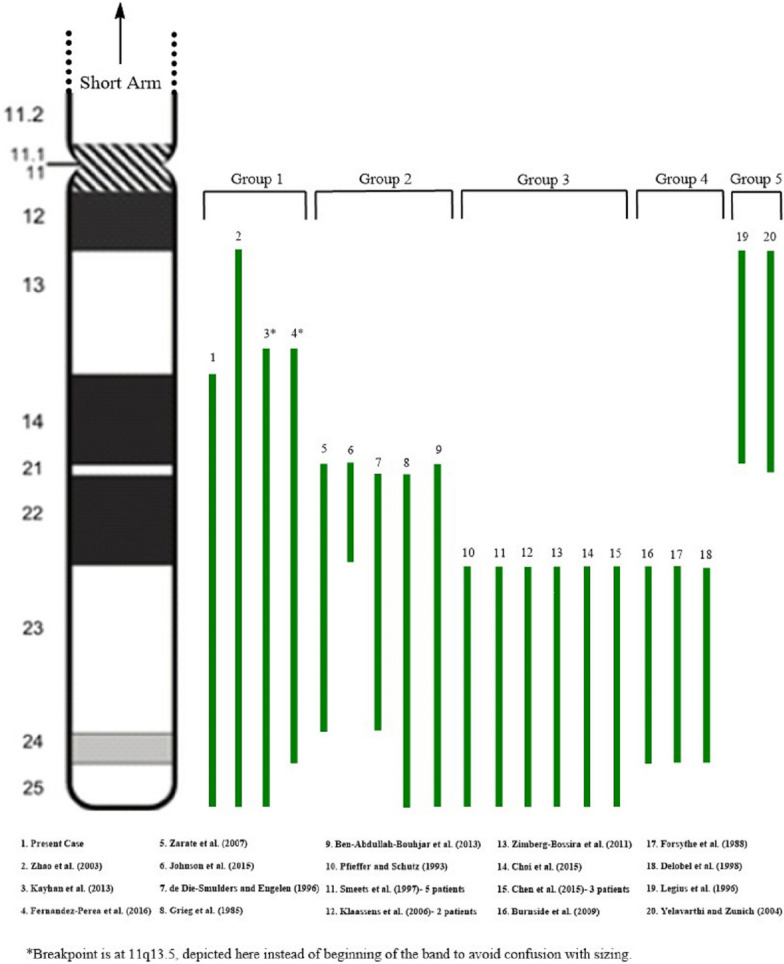

**Supplementary Information:**

The online version contains supplementary material available at 10.1186/s13039-022-00595-0.

## Introduction

Partial trisomy of the long arm of chromosome 11 is a rare cytogenetic abnormality first reported as a clinical entity in 1972 [1] and described in detail in 1977 [2]. The main features of partial trisomy 11q syndrome include: pre/postnatal growth retardation, developmental delay, intellectual disability, and dysmorphic craniofacial features. Craniofacial features may include microcephaly, hypertelorism, up-slanting palpebral fissures, low-set ears, short nose, high arched or cleft palate, and micrognathia. Some affected individuals showe additional findings, such as structural malformations of the heart, skeletal and limb anomalies, congenital diaphragmatic hernia, and hypotonia. Undescended testis and small penis are found in male patients. Clinical findings are thought to be correlated with the size and genomic contents of the duplicated region [3–5].

The majority of previously reported cases are the result of unbalanced translocations that involve partial monosomy of a partner chromosome. Delineation of genotype and phenotype in these cases is extremely difficult because the clinical features of those cases were due to the combined effects of both trisomy 11q and deletions of the partner chromosomes. Cases that occur without significant involvement of other chromosomes are therefore valuable in order to understand the clinical consequences of trisomy 11q. These types of cases have been referred to as “pure” trisomy 11q.

There are three different types of “pure” trisomy 11q cases found in the literature. The first are interstitial duplications of 11q, which are certain to not have any interference from other chromosomes. To our knowledge, there are eleven reported patients with such abnormalities [6–16]. Second are cases that involve translocations resulting in trisomy 11q and monosomy of the short arm of an acrocentric chromosome, in which genetic material in the deleted regions has limited impact on the clinical features. We identified five patients with this type [17]. Third are cases of unbalanced translocations resulting in 11q trisomy with no deletions or submicroscopic deletions of the partner chromosomes. These cases are considered as “pure” trisomy 11q because there is no or very minimum dosage effect from other chromosomes. We have identified ten patients with this type in the literature [3–5, 18–21].

Here, we report the molecular cytogenetic findings and clinical features of a 16-year-old patient with a large sized (56 Mb) trisomy 11q14.1-qter and review all reported cases of “pure” trisomy 11q.

## Methods

### Case presentation

A 16-year-old male presented at our genetics clinic for a routine follow-up. He has been a patient at our medical center since birth, and has received care at our neurology, cardiology, endocrinology, gastrointestinal, orthopedics, and ophthalmology departments due to multiple congenital anomalies.

The patient was born by C-section at 36-week gestational age after a pregnancy complicated by oligohydramnios. He had multiple congenital heart defects including atrial septal defect, ventricular septal defect, and pulmonary stenosis. He also had dysmorphic craniofacial features with microcephaly, large anterior fontanelle, low-set ears, malformed auricles, micrognathia, and high-arched palate. Congenital hip dislocation of the right hip was also noticed. Hearing test was performed and revealed sensorineural hearing loss.

During his infancy and childhood, he was found to have global developmental delays, speech delay, seizures, and increased tonicity of the extremities. Left optic nerve hypoplasia was diagnosed by an ophthalmologist. Hypopituitarism was identified by an endocrinologist and treatment with testosterone and levothyroxine was started. He was referred to gastroenterology for poor weight gain. Joint stiffness and pain were noticed on physical evaluation. Bone age was evaluated when the patient was 5 years old due to clinical concern for short stature, the results revealed a delayed bone age with greater than 3 standard deviations below the mean. Multiple surgeries were performed to repair congenital heart defects, hearing loss, and hernia.

Currently, at the age of 16, the patient is extremely small and thin for his age appearing to be age 4–5. He has short stature, with height of 116 cm (45.67 in), at zero percentile. He has deficiencies in both growth hormone and testosterone. His weight fluctuates between 13–16 kg and has been hospitalized twice for weight loss concerns. Hypogonadism is apparent with Tanner 1 genitalia and unpalpable testes. He has a mild degree of levoscoliosis of the lumbar spine. He has also been diagnosed with seizure disorder, growth hormone deficiency, hypothyroid, and failure to thrive. His ears are small and low set. Micrognathia is present and has led to feeding issues. Mild hypertonia is noted with limited extension of limbs. He has indications of a defective immune system with recurrent ear infections, urinary tract infections, and went into septic shock following a dental surgery.

Patient is the only child between his parents and the only person with chromosomal anomaly within the family. Mom is reported to have scoliosis and arthritis. She also has a history of two first trimester miscarriages with this patient’s father. Father is reportedly healthy. Patient has two maternal half-sisters who are both healthy. He has two paternal half-bothers, with one of them has speech delay and ADHD. Parental chromosome analysis was not performed because the family denied further testing.

### Cytogenetic analysis

Routine chromosome analysis was performed using the standard laboratory protocol [22]. Chromosomes at the metaphase stage were prepared from phytohemagglutinin (PHA) stimulated lymphocyte culture. Karyotype analysis was conducted by the G-banding technique with 550-band resolution. The cytogenetic abnormalities were described according to the International System for Human Cytogenetic Nomenclature (ISCN) 2016.

### Chromosomal microarray analysis

DNA samples were extracted from peripheral blood using Maxwell RSC Instrument and Maxwell RSC Blood DNA Kit (Promega, 2800 Woods Hollow Road, Madison, WI 53711 USA) following manufacturer’s protocol. Chromosome microarray (CMA) was performed using the illumina Human HD CytoSNP 850 V1.2 array (5200 Illumina Way, San Diego, CA 92122 USA), which includes over 850 K single nucleotide polymorphisms (SNPs) probes to detect copy number variation (CNV) and loss of heterozygosity (LOH) in the human genome. The procedures and array scanning were performed according to the manufacturer’s instruction with minus adjustment. Data were analyzed with software Bluefuse multi V4.5. The reportable segment was restricted to regions of any size with 10 or more consecutive probes that differed significantly from the expected normalized diploid values and overlaps with one OMIM annotated gene at least.

### Review methods

Literature for the review portion of the discussion was selected using the search engine PubMed. The key words “trisomy 11q” and “11q duplication” were the primary search terms. “Trisomy 11q” yielded 61 results and “11q duplication” yielded 119 results, all abstracts of which were read to find cases that did not include interaction with other chromosomes. When a case was determined to have a “pure” trisomy 11q, all of the references used in that case’s bibliography would be investigated. All “pure” trisomy 11q cases are detailed in Tables 1–5 in Additional Files.

## Results

**Fig. 1:**
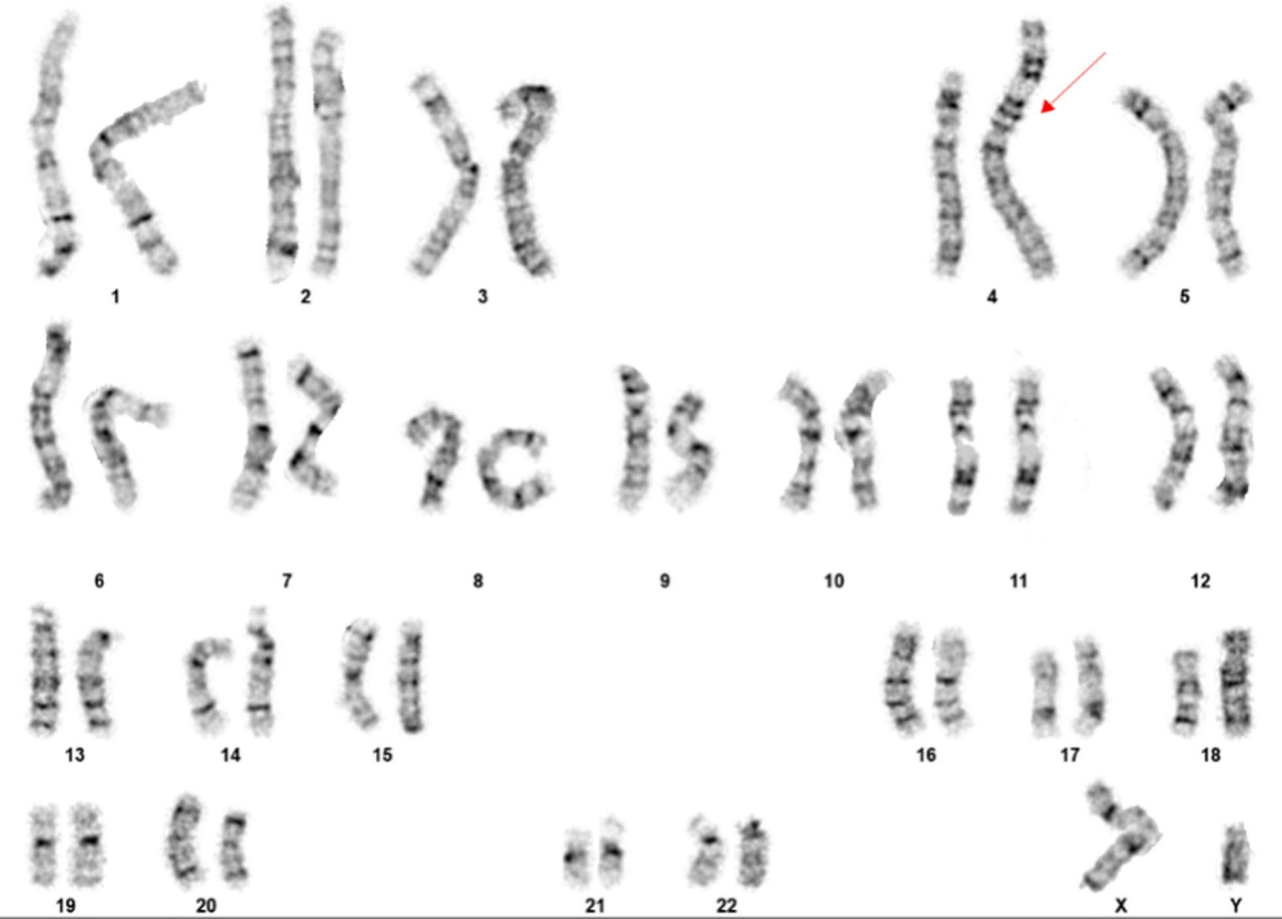
Routine chromosome analysis showed an abnormal karyotype result 46,XY,der(4)t(4;11)(p16.3;q14.1) in this patient, resulting in partial trisomy of 11q

Conventional cytogenetic study revealed an abnormal male chromosome complement in all cells examined with a derivative chromosome 4 caused by an unbalanced translocation between chromosomes 4 and chromosome 11. The breakpoints were at bands 4p16.3 and 11q14.1 (Fig. 1). The karyotype was designated as 46,XY,der(4)t(4;11)(p16.3;q14.1). This rearrangement resulted in partial trisomy of the long arm of chromosome 11 (11q14.1-qter) and a terminal deletion of the short arm of chromosome 4 (4p16.3-pter).

**Fig. 2:**
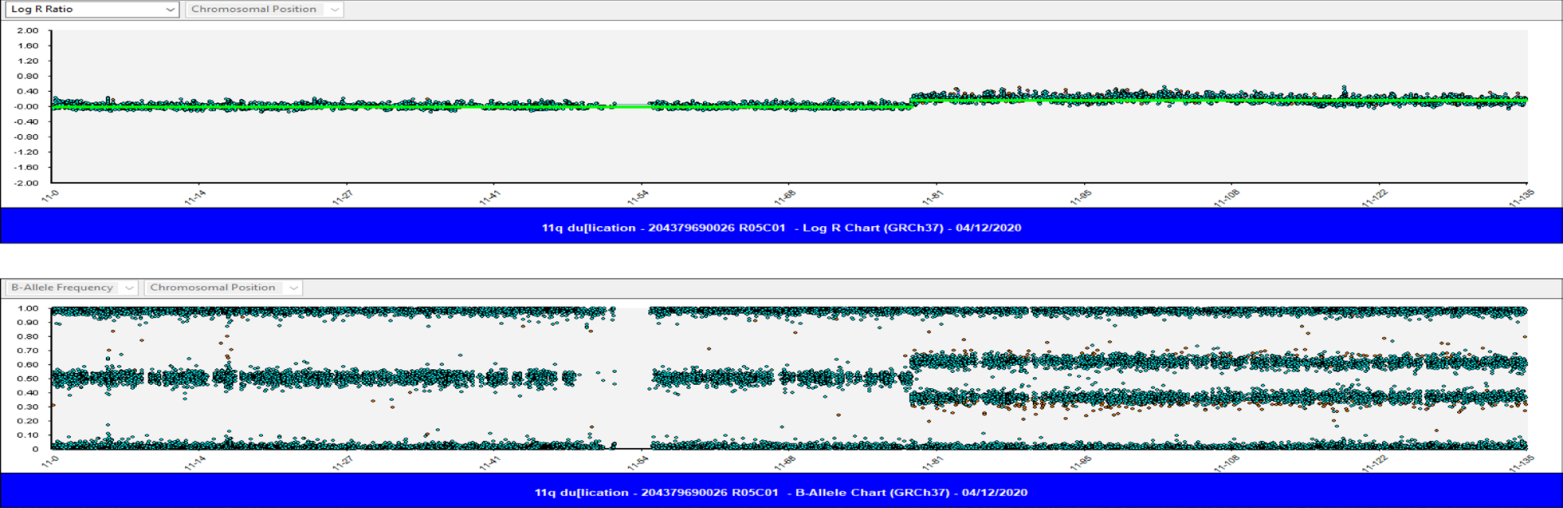
SNP array analysis utilizing illumina Human HD CytoSNP 850 V1.2 platform showed a 56 Mb duplication in region 11q14.1-qter (chr11: 78,694,515–134,934,063, GRCh37)

CMA analysis was performed to further characterize the chromosomal abnormalities described above. The test results revealed a 56 Mb copy number gain of chromosome 11q14.1-qter and a 874 Kb copy number loss of chromosome 4p16.3-pter. CMA results were described as: arr[GRCh37] 4p16.3(49450_923261) × 1, 11q14.1q25(78694515_134934063) × 3 Fig. 2). CMA results are consistent with cytogenetics findings. Additionally, the 11q duplicated region contains ~ 760 RefSeq genes and 232 OMIM annotated genes; the 4p deleted region is overlapped with 25 RefSeq genes and 8 OMIM annotated genes.

**Discussion**

Partial trisomy 11q is a very rare chromosomal disorder that is characterized by growth retardation, developmental delay/intellectual disability, and distinctive craniofacial abnormalities. The clinical features may vary, and are usually associated with the location, size, and genomic consents of the chromosome regions involved. There are limited number of partial trisomy 11q cases reported in the literature, with many of them occur along with partial monosomy of another chromosome due to an unbalanced translocation. For this reason, patients present with additional signs and symptoms other than partial trisomy 11q syndrome. Diagnosis of partial 11q trisomy is established based on results of routine chromosomal analysis; chromosomal microarray analysis (CMA) provides additional information for genotype–phenotype correlation.

In this study, we reported a 16-year-old male with core features of trisomy 11q due to an unbalanced translocation involving chromosomes 4 and 11, culminating a derivative chromosome 4 in this patient. Using high resolution CMA, we further characterize this unbalanced rearrangement. The CMA results revealed a 56 Mb duplication of chromosome 11q14.1-qter and a 874 Kb terminal deletion of 4p16.3-pter. There are 25 RefSeq genes overlapped with the 4p deleted region, with four of them are OMIM morbid genes. Among these four genes, mutations in the ZNF141 and CPLX1 genes are associated with autosomal recessive postaxial polydactyly type A6 and developmental and epileptic encephalopathy-63, respectively. This patient does not show symptoms related to these disorders. Biallelic pathogenic variants in the PIGG gene have been reported in patients with autosomal recessive intellectual developmental disorder-53. However, most of mutations reported in the literature are predicted to cause loss of function of PIGG. Large-sized duplications involving the PIGG gene have not been reported, to our knowledge. Of note, the PDE6B gene is also included in the deleted region; mutations in this gene can cause autosomal dominant congenital stationary night blindness-2 or autosomal recessive retinitis pigmentosa-40. However, partial or entire gene deletions of PDE6B were only found in patients with retinitis pigmentosa, the autosomal recessive form of PDE6B-related disorders. In addition, terminal deletions of the short arm of chromosome 4 are associated with Wolf-Hirschhorn syndrome (WHS), a contiguous gene deletion syndrome with main features of craniofacial anomalies, growth deficiency, developmental delay/intellectual disability of variable degree, and seizures. The sizes of 4p deletions are variable in patients, with two critical regions for WHS have been proposed. The first one spanned ~ 145 kb and locates about 2 Mb from the telomere [22]. The second critical region for WHS is a 300- to 600-Kb interval on 4p16.3 between D4S3327 and D4S98-D4S168, which is ~ 1.9 Mb to the telomere [23]. The 4p deletion identified in our patient does not fall in either of these critical regions. We also notice that deletion of this region has not been documented in the general population, nor has it been reported in the literature. Taken together, we believe that the clinical significance of the 4pter deletion is uncertain at this point. In contrast, chromosome 11q14.1q25 duplicated region encompasses more than 700 RefSeq genes, and duplication of this region is known to cause trisomy 11q syndrome. Thus, we consider the phenotype observed in our patient is predominantly caused by partial trisomy 11q. To our knowledge, this is the third largest trisomy 11q to be reported, with a unique cytogenetic finding of the unbalanced translocation t(4;11).

**Fig. 3:**
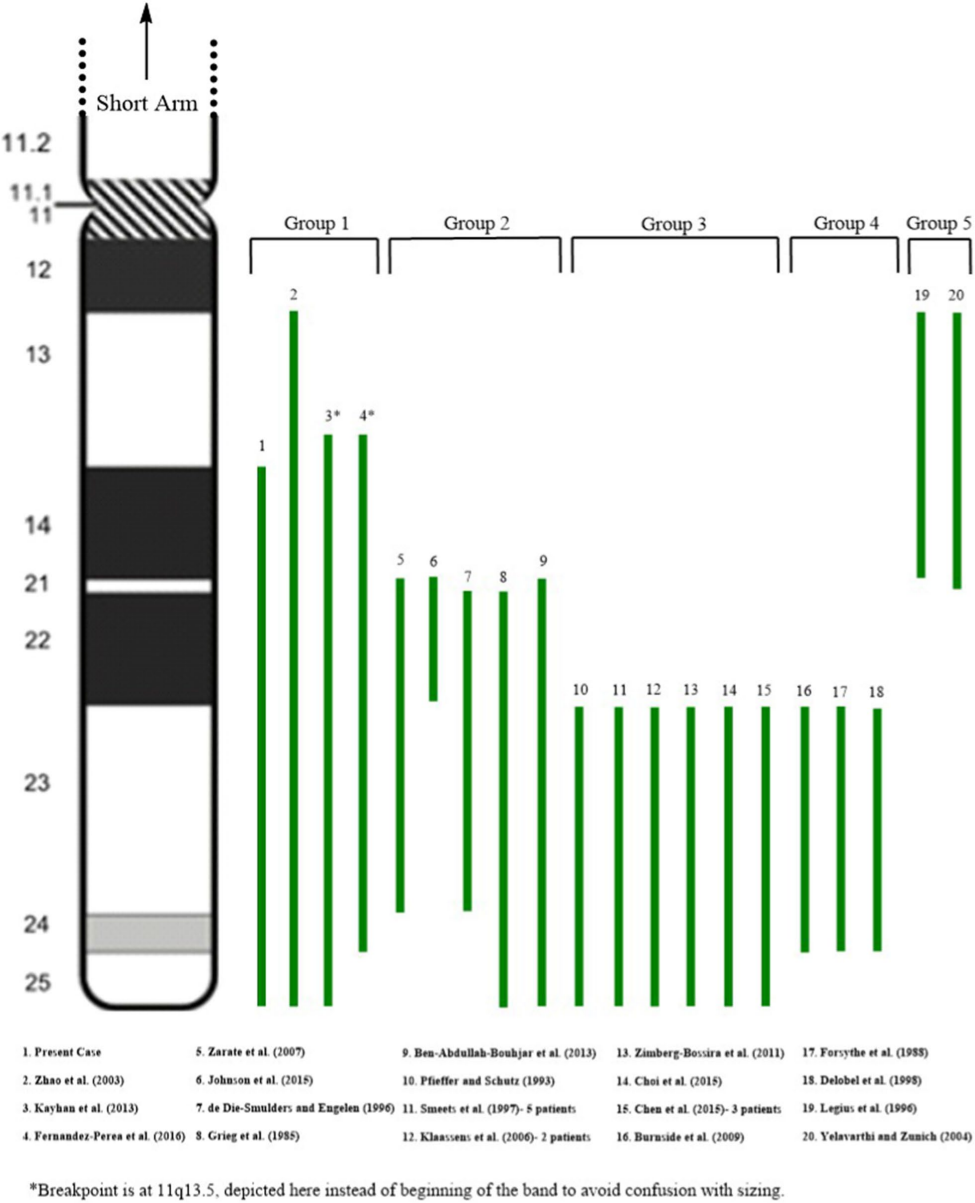
Schematic illustration of the long arm of chromosome 11, comparing the size and location of previous 11q duplications reported in Tables 1–5. Idiogram shows the long arm of chromosome 11 at 400-band resolution. Refer to Tables 1–5 for precise description of duplicated segments

“Pure” trisomy 11q cases are described as cases with minimal or no involvement of another chromosome. Few cases of this type have been reported and they provide a unique opportunity for evaluating the clinical consequences of increased dosage of genes on 11q. So far, 26 patients with this type of abnormality have been described in 19 case reports. These patients show core features of trisomy 11q syndrome plus additional findings related to multiple factors, including different types of chromosomal abnormalities, sizes and genomic contents of the duplicated regions. We reviewed conventional cytogenetic and/or molecular genetics findings as well as clinical findings from these cases and compared to the results from current study. We determined that the partial trisomy 11q identified in our patient was one of a few large sized duplications that involves almost the entire long arm of chromosome 11. We also summarized laboratory findings and phenotypes of the reported cases. We divided the reported cases into five categories based on the common chromosomal regions involved: 11q14-q24, 11q22, 11q23-qter, 11q23-q24, and 11q13-q14 (Fig. 3). Some patients have trisomy 11q spanning multiple regions. For the convenience of comparison, we described the cytogenetic results from different studies using 400-band level. Details of the partial trisomy 11q cases are summarized in Tables 1–5 in Additional Files.

Table 1 in Additional Files describes four trisomy 11q cases that involves almost the entire long arm of chromosome 11, with a common region of 11q14-q24. This group includes our patient and a prenatal case with pregnancy termination in the second trimester. Two cases showed pure duplication of 11q, and two other cases have unbalanced translocations. As expected, these patients presented more severe features compare to those who has relatively small-sized trisomy 11q in other groups. All applicable patients had short stature/growth retardation, developmental delay (DD), dysmorphic facial features, congenital heart defects (CHD), seizures, urogenital and extremity abnormalities. Common facial features include microcephaly, vision abnormalities, abnormal shaped or low-set ears, short nose with flat nasal bridge, micrognathia, and high-arched palate. Hands and feet anomalies, i.e. brachydactyly, hypoplastic nails, and pes planus, are found in all three postnatal cases. Renal agenesis or hypoplastic kidney are observed in two female patients; micropenis and cryptorchidism are identified in our patient, the only male patient in this group. We consider that the large number of genes and dosage effects of these genes are associated with the relatively severe phenotypes observed in this group. In addition, patients in this group may present “mixed” and combined phenotypes found in patients from other groups who only have partial trisomy 11q.

Table 2 in Additional Files describes five cases with variable sizes of trisomy 11q, with the minimum overlapping region of 11q22. In this group, two patients (# 6 and 7 in Fig. 3) were reported in the literature at age of 46 and 50, notable as the majority of trisomy 11q patients do not survive into adulthood. Two other patients (# 8 and 9 in Fig. 3) carry similar trisomies (11q22-qter and 11q21-qter, respectively) resulting from unbalanced translocations involving different partner chromosomes. Although trisomy 11q with different sizes are found in this group, we identified common features among these patients including epicanthal folds, up-slanting palpebral fissures, hypertelorism, micrognathia, and extremities deformations (e.g. clinodactyly and syndactyly). Microcephaly and developmental delays were observed in three patients with large-sized trisomy 11q in this group. In general, patients in this group showed relatively mild clinical features than subjects in other groups.

Table 3 in Additional Files describes multiple cases with trisomy 11q involving an identical region from 11q23 to 11qter. A total of 12 patients were included in this group, making this group with the largest number of subjects. All of these were inherited, either paternally or maternally. In particular, Smeets et al. [17] described 5 patients with trisomy 11q caused by an unbalanced translocation between chromosome 11 and 13 from the same family. In the remaining cases, trisomy 11q are due to the pure duplication or unbalanced translocations with different partner chromosomes. We noticed that trisomy 11q23-qter is a recurrent abnormality in this group of patients. Not surprisingly, these patients share similar features, such as mental retardation (MR), hypotonia, dysmorphic facial features, congenital heart defects (CHD), upper airway malformations, congenital diaphragmatic hernia (CDH) and/or inguinal hernia, and micropenis in male patients. Dysmorphic facial features are similar to those described in patients with large-sized trisomy 11q (group 1). Of note, these clinical findings represent the core features of partial trisomy 11q syndrome. Furthermore, we found that the breakpoint 11q23 identified in this group patients is the same as that observed in the recurrent translocation t(11;22)(q23;q11.2). Translocation t(11;22) is the most common recurrent non-Robertsonian constitutional abnormality described in human; carriers with the balanced form have an increased risk to produce offspring with Emanuel syndrome, usually caused by a supernumerary chromosome–47,XX or XY, + der(22)t(11;22)(q23;q11.2). This is an unbalanced rearrangement resulting in trisomy 11q23-qter and trisomy 22 pter-q11.2. Emanuel syndrome is characterized by significant developmental delay and mental retardation, microcephaly, ear anomalies, cleft or high-arched palate, micrognathia, heart malformations, renal defects, as well as small penis and cryptorchidism in males. Kurahashi et al. [25, 26] suggested that the palindromic AT-rich repeats (PATRRs)- mediated chromosomal translocation was the possible mechanism of this recurrent rearrangement [24, 25]. Interesting enough, we found that patients with Emanuel syndrome share very similar features with subjects in this group. This phenotypical similarity is likely due to an identical cytogenetic anomaly, trisomy 11q23-qter. Although there are a large number of genes located in this region, previous studies provide evidence that some genes possibly contribute to the key features described above. Ben-Abdallah-Bouhjar et al. [19] hypothesized that the NCAM1, DRD2, THY1, and GRIK4 genes at 11q23.2-q23.3 region may be responsible for intellectual disability, short stature, dysmorphic facial features, hypotonia, and congenital heart defects. Three genes located at 11q24.2 region, ROBO3, ROBO4, and CDON, are possible candidate genes for congenital diaphragmatic hernia [5].

Table 4 in Additional Files describes three cases with trisomy of the 11q23 -11q24 region. All these cases are causes by intra-chromosomal rearrangement of 11q with apparently identical break points. Compared to the abnormalities described in group 3, the duplicated region found in this group share the same break point 11q23 at the proximal end, but has a different break point 11q24 at the distal end. In other words, patients in this group have a smaller sized trisomy 11q. Correspondingly, patients in this group have overlapping but milder features than patients with trisomy 11q23-qter. Common features in this group include mental retardation, microcephaly, up-slanting palpebral fissures, strabismus, ear abnormalities, and extremity anomalies. In contrast, cleft or high-arched palate, micrognathia, heart malformations, renal and urogenital defects were not identified. Overall, we consider this group as a variant of trisomy 11q23-qter, but with milder features.

Table 5 in Additional Files describes two cases of trisomy 11q that resulted from interstitial duplications in the proximal region of 11q13-q14. The duplicated segment in one case is 11q13.3-q14.2, and the other has trisomy 11q13.5-q21. Both patients have unspecific craniofacial features and skeletal anomalies, as well as mild to moderate developmental delay. More cases are needed to determine clinical features of trisomy of proximal 11q region.

## Conclusion

In summary, we reported the clinical characteristics of a patient carrying a rare 11q trisomy, resulting from the unbalanced translocation t(4;11)(p16.3;q14.1). This patient presents with multiple congenital anomalies predominately caused by trisomy 11q. It is the third largest trisomy 11q to be reported, with a unique cytogenetic finding of t(4;11). To have a better understanding and delineation of trisomy 11q syndrome, we compared our case with the reported cases of “pure” trisomy 11q in the literature. We divided the reported cased into five groups based on the common cytogenetic regions involved and summarized clinical features of each group. We observe that trisomy 11q23-qter and its variant (11q23-q24) are recurrent and the most common abnormality identified in patients with “pure” trisomy 11q. Phenotypes related to this abnormality represent the core features of trisomy 11q syndrome, including mental retardation/developmental delay, craniofacial features, congenital heart defects, upper airway malformations, congenital diaphragmatic hernia and/or inguinal hernia, as well as renal and urogenital defects. Patients with large sized duplications involving both proximal and distal 11q present a severe phenotype involving multisystemic congenital anomalies. In contrast, duplication of proximal 11q region is associated with relatively mild features.

## Supplementary Information

**Additional file 1.** Table 1. Summaries of trisomy 11q cases from group 1.

**Additional file 2.** Table 2. Summaries of trisomy 11q cases from group 2.

**Additional file 3.** Table 3. Summaries of trisomy 11q cases from group 3.

**Additional file 4.** Table 4 Summaries of trisomy 11q cases from group 4.

**Additional file 5.** Table 5. Summaries of trisomy 11q cases from group 5.

## Data Availability

All data generated or analyzed during this study are included in this published article.
