# Supplementary material for: Molecular cytogenetic characterization of partial trisomy of the long arm of chromosome 11 in a patient with multiple congenital anomalies
Source: Mol Cytogenet. 2022 Apr 19;15:17. doi: 10.1186/s13039-022-00595-0 (PMC9019979; doi:10.1186/s13039-022-00595-0)
Supplement: Supplementary file 1 — Additional file 1. Table 1. Summaries of trisomy 11q cases from group 1. [file 13039_2022_595_MOESM1_ESM.docx]

| **TABLE 1** | *Zhao et al. (2003)* | *Kayhan et al. (2013)* | *Fernandez-Perea et al. (2017)* | Present Case |
| --- | --- | --- | --- | --- |
| **Figure 3 reference number** | **2** | **3** | **4** | **1** |
| **Number of patients** | 1 (Patient 3) | 1 | 1 (fetus) | 1 |
| **Cytogenetics and molecular genetics findings** | 46,XX,add(11)(q25).ish dup(11)(q25q13)(wcp11+) | 46,X,der(X)(Xqter → Xp22.33::11q13.5 → 11qter).ish der(X)(DXYS129+, D11S1037+).arr 11q13.5q25(76,601,607- 134,926,021)x3 dn | 46,XX,dup(11)(q13.5q24).ish dup(11)(MLL++,D11S1037+) | 46,XY,der(4)t(4;11)(p16.3;q14.1) |
| **Duplicated segment** | q13–q25 | q13.5–qter | q13.5-q24 | q14.1-qter |
| **Partner chromosome** | none | X chromosome | none | chromosome 4 |
| **Most recent age at examination/sex** | 5 years/F | 19 months/F | terminated pregnancy at 23+4 weeks of gestation | 16 years/M |
| **Short stature/growth retardation** | + | - | NR | + |
| **Microcephaly** | + | - | NR | + |
| **Eyes** | myopia, coloboma of iris | lacrimal duct stenosis, periorbital edema (only at birth) | NR | left optic nerve hypoplasia |
| **Ears** | cupped ear, left preauricular sinus | abnormal ears | NR | low set, malformed auricles, preauricular cyst, hearing loss |
| **sNose** | flat nasal brige, small nose with bulbous tip | short nose | NR | - |
| **Mouth** | microstomia, high arched palate | long philtrum, thin upper lip, high arched palate | NR | high arched palate |
| **Micrognathia** | + | + | NR | + |
| **Congenital heart defects** | ASD | PDA | NR | ASD, VSD, pulmonary stenosis |
| **Upper airway malformation** | malformed epiglottis, tracheomalacia | - | NR | + |
| **Skeletal anomalies** | short limbs, asymmetric leg length | separated proximal and distal sites of right clavicle | NR | delayed bone age, scoliosis, hip dislocation, joint pain and stifness |
| **Extremities** | brachydactyly, right single palm crease | hypoplastic nails | NR | pes planus |
| **Urogenital anomalies** | NR | hypoplastic left kidney, hypoplastic labium minor, proteinuria | NR | micropenis, cryptorchidism |
| **Mental retardation/development delay** | + | + (mild) | NR | + |
| **Hypertonia** | NR | NR | NR | + |
| **Hypotonia** | + | + (only at birth) | NR | - |
| **Seizures** | + | + | NR | + |
| **Other** | round face, recurrent otitis media, tracheostomy dependent, plagiocephaly, feeding intolerance, gastro-esophageal reflux requiring gastro-jejunostomy tube, pregnancy complicated by toxemia | large forehead, cavum ceptum pellicidum formation, hypoplasia of third left toenail, wheezy respiration pattern | congenital diaphragmatic hernia with cardiomediastinal shift and bilateral lung hypoplasia | hypopituitarism, recurrent infections, hernia, low anterior fontanelle |

NR: Not Recorded; VSD: ventricular septal defect; PVS: pulmonary valve stenosis; PDA: patent ductus arteriosus; ASD: atrial septal defect.

- Means no abnormal findings; + Means abnormal findings
