# Supplementary material for: Molecular cytogenetic characterization of partial trisomy of the long arm of chromosome 11 in a patient with multiple congenital anomalies
Source: Mol Cytogenet. 2022 Apr 19;15:17. doi: 10.1186/s13039-022-00595-0 (PMC9019979; doi:10.1186/s13039-022-00595-0)
Supplement: Supplementary file 2 — Additional file 2. Table 2. Summaries of trisomy 11q cases from group 2. [file 13039_2022_595_MOESM2_ESM.docx]

| **TABLE 2** | *Zarate et al. (2007)* | *Johnson et al. (2015)* | *de Die-Smulders and Engelen (1996)* |
| --- | --- | --- | --- |
| **Figure 3 reference number** | **5** | **6** | **7** |
| **Number of patients** | 1 | 1 (Patient 277-10) 4 other family members stated to have similar trisomy, but no other data provided | 1 |
| **Cytogenetics and molecular genetics findings** | 46,XY,dup(11) (q14.1q21).ish dup(11)(q14.1q21)(wcp11+,CCND1+) a copy number gain of 11q21 to 11q23.1, ~16.2 Mb | a 7.5Mb duplication at chromosome 11q21 to11q22.3 (between rs11021461 and rs10488755) | 46,XX, dir dup(11)(pter→q23::q22→qter) |
| **Duplicated segment** | q21–q23.1 | q21-q22 | q22–q23 |
| **Partner chromosome** | none | none | none |
| **Most recent age at examination/sex** | 4 years/M | 46 years/M | 50 years/F |
| **Short stature/growth retardation** | - | NR | <3rd centile, but measurement difficult |
| **Microcephaly** | - | NR | 3rd centile |
| **Eyes** | epicanthal folds, short palpebral fissures | strabismus, saccadic pursuit, horizontal gaze palsy | prominent eyes, upward slanting palpebral fissures, hypertelorism, slight ptosis |
| **Ears** | posteriorly rotated ears, right Darwinian tubercle, | hearing impairment | large, protruding |
| **Nose** | flat nasal bridge, mildly anteverted nares, small | NR | beaked nose |
| **Mouth** | NR | NR | lower lip eversion, wide mouth, short and flat philtrum |
| **Micrognathia** | mild | NR | prognathia |
| **Congenital heart defects** | NR | NR | NR |
| **Upper airway malformation** | NR | NR | NR |
| **Skeletal anomalies** | NR | thoracic deformation | severe kyphoscoliosis, maxillary hypoplasia, spina bifida occulta |
| **Extremities** | clinodactyly (5th finger) | foot deformation, lower limb muscle wasting | underdeveloped arms, relatively large and fleshy hands, deep palmar furrows, hyperextensible fingers, bilateral drop-feet, severe lower leg edema |
| **Urogenital anomalies** | NR | NR | NR |
| **Mental retardation/development delay** | - | + (mild) | + |
| **Hypertonia** | NR | moderate spasticity of  the lower limbs | + |
| **Hypotonia** | NR | NR | NR |
| **Seizures** | NR | NR | NR |
| **Other** | frequent respiratory and ear infections, brachycephaly, flat facial profile, articulation problems | cerebellar dysmetria in the upper limbs, cerebellar atrophy with  periventricular white matter hyperintensities by MRI, vibration sense abolished up to hips, increased reflexes with ankle clonus and positive Hoffman and Babinski signs | feeding problems, small face, flat umbilicus |

**TABLE 2-continued**

|  | *Grieg et al. (1985)* | *Ben-Abdullah-Bouhjar et al. (2013)* |
| --- | --- | --- |
| **Figure 3 reference number** | **8** | **9** |
| **Number of patients** | 1 | 1 |
| **Cytogenetics and molecular genetics findings** | 46,XY,der(9)t(9;11)(p24;q22). | 46,XY,der(7)t(7; 11)(p22;q21)pat , |
| **Duplicated segment** | q22-qter | q21-qter |
| **Partner chromosome** | chromosome 9 | chromosome 7 |
| **Most recent age at examination/sex** | 7 months/M | 3 years/M |
| **Short stature/growth retardation** | <3rd centile | + |
| **Microcephaly** | <3rd centile | + |
| **Eyes** | anti-mongoloid slanted palpebral fissures, blindness | hypertelorism, up-slanting palpebral fissures |
| **Ears** | low set and posteriorly rotated, scant eyebrow hair | low set |
| **Nose** | NR | beaked nose |
| **Mouth** | highly arched palate | highly arched palate, retracted lip |
| **Micrognathia** | hypoplastic mandible | + |
| **Congenital heart defects** | - | - |
| **Upper airway malformation** | NR | - |
| **Skeletal anomalies** | NR | hip dislocation |
| **Extremities** | clinodactyly (5th finger on left) and syndactyly (4th and 5th finger on right), single palmar crease, prominent heels | NR |
| **Urogenital anomalies** | micropenis with marked chordee, ambiguous genitalia, perineal hypospadias, bifid scrotum with high testes | small penis |
| **Mental retardation/development delay** | + | + |
| **Hypertonia** | NR | muscular hypertonia with pyramidal syndrome (neonatal period) |
| **Hypotonia** | poor head control, cannot sit unsupported | + (neonatal period) |
| **Seizures** | + | + |
| **Other** | feeding problems, intermittent diarrhea, umbilical hernia, large anterior fontanelle, closed posterior fontanelle, low hairline, | congenital inguinal hernia, hiatal hernia, structural asymmetry of face, frequent infections, corpus callosum agenesis, cerebellum atrophy, and simple cerebral gyration. |

NR: Not Recorded; VSD: ventricular septal defect; PVS: pulmonary valve stenosis; PDA: patent ductus arteriosus; ASD: atrial septal defect.

- Means no abnormal findings; + Means abnormal findings
