# Supplementary material for: Molecular cytogenetic characterization of partial trisomy of the long arm of chromosome 11 in a patient with multiple congenital anomalies
Source: Mol Cytogenet. 2022 Apr 19;15:17. doi: 10.1186/s13039-022-00595-0 (PMC9019979; doi:10.1186/s13039-022-00595-0)
Supplement: Supplementary file 3 — Additional file 3. Table 3. Summaries of trisomy 11q cases from group 3. [file 13039_2022_595_MOESM3_ESM.docx]

| **TABLE 3** | *Pfeiffer and Schutz (1993)* | | *Smeets et al. (1997)* | | *Klaassens et al. (2006)* | | |  |
| --- | --- | --- | --- | --- | --- | --- | --- | --- |
| **Figure 3 reference number** | **10** | | **11** | | **12** | | |  |
| **Number of patients** | 1 | | 5 (Patients 1-5) | | 2 siblings | | |  |
| **Cytogenetics and molecular genetics findings** | 46,XY,dup(11)(q23qter), mat | | 46,XY,der(13)t(11;13)(q23;p13) mat or pat | | 46,XY,der(12)t(11;12)(q23;q24) mat, ~19Mb duplication of 11q23.2qter and a <0.5Mb deletion of 12qter | | |  |
| **Duplicated segment** | q23–qter | | q23-qter | | q23-qter | | |  |
| **Partner chromosome** | none | | chromosome 13 | | chromosome 12 | | |  |
| **Most recent age at examination/sex** | 7 months/M | | Patient 1- 25 years/M, Patient 2- 10 years/M, Patient 3- 9 years/M, Patient 4- 6 months/M, Patient 5- 10 months/M | | Patient 1- 2 years/M, Patient 2-7 days/M | | |  |
| **Short stature/growth retardation** | + | | -(3), NR(2) | | -(2) | | |  |
| **Microcephaly** | + | | -(3), NR(2) | | -(2) | | |  |
| **Eyes** | hypotelorism, epicanthus inversus, long eyelashes | | up-slanted palpebral fissures (3), strabismus (1), telecanthus (1), NR(2) | | hypertelorism (1), metoptic ridge (1), upslanting of the eyes with telecanthic folds (1), short palpebral fissures (1) | | |  |
| **Ears** | large and prominent | | low-set (2), dysplastic (1), NR(2) | | low set (1), NR(1) | | |  |
| **Nose** | short nose | | short nose (4), bulbous tip (1), full tip (1), NR(1) | | short nose (2), broad, flat, upturned nose (1) | | |  |
| **Mouth** | small mouth, high arched palate | | long philtrum (3), high arched palate (3), narrow palate (2), cleft palate (2), retracted lower lip (2), everted lower lip (1) | | open mouth appearance (1), prominent philtrum (1) | | |  |
| **Micrognathia** | + | | microretrognathia (5) | | +(2) | | |  |
| **Congenital heart defects** | ASD | | ASD (2), aortic coarctation (1), unspecified heart defect (1), NR(2) | | VSD (1), NR(1) | | |  |
| **Upper airway malformation** | NR | | Pierre-Robin sequence (2), NR(3) | | Pierre-Robin sequence (1), NR(1) | | |  |
| **Skeletal anomalies** | - | | hip dislocation (3), scoliosis (1), bilateral Perthes disease (1), bilateral hip dysplasia (1) | | NR (2) | | |  |
| **Extremities** | - | | club feet (1), clinodactyly (1), NR(4) | | prominent heels (2), hypoplastic toenails (1) | | |  |
| **Urogenital anomalies** | short penis, shallow scrotum | | micropenis (4), undescended testicles (1), NR(1) | | micropenis (2), unilateral hydronephrosis (1) | | |  |
| **Mental retardation/development delay** | + | | +(2) severe, +(1) moderate to severe, +(1) moderate, NR(1) | | + (1) NR (1) | | |  |
| **Hypertonia** | NR | | -(1), +(2), NR(2) | | NR (2) | | |  |
| **Hypotonia** | NR | | +(4) neonatal, NR(1) | | +(2) | | |  |
| **Seizures** | NR | | +(3), NR(2) | | NR (2) | | |  |
| **Other** | congenital inguinal hernia, minor dilation of lateral ventricles, septum pellucidum cyst, flattened occiput, pilonidal dimple | | Recurrent upper airway infections (3), Pierre-Robin sequence (2), unspecified respiratory problems leading to death (1), congenital inguinal hernia (1) | | congenital diaphragmatic hernia (1), pulmonary hypoplasia (1), high anterior hairline (1), short/broad neck (1), loose skin and subcutaneous tissue (1), recurrent pneumothorax (1) | | |  |
|  | | | | |  |  |  |  |
| **TABLE 3-continued** | | *Zimberg-Bossira et al. (2011)* | | *Choi et al. (2015)* | | *Chen et al. (2014)* | | |
| **Figure 3 reference number** | | **13** | | **14** | | **15** | | |
| **Number of patients** | | 1 | | 1 | | 3 siblings (Patients 1-3) | | |
| **Cytogenetics and molecular genetics findings** | | 46, XY,der(21)t(11;21)(q23.1;q22.1) pat | | 46,XY,der(22) t(11;22)(q23.3;q13.3)mat | | 46,XY(XX),der(10)t(1o;11)(q26;q23) pat, a 15.1Mb duplication of 11q23.3qter and a 470Kb deletion of 10qter | | |
| **Duplicated segment** | | q23.1–qter | | q23.3-qter | | q23.3-qter | | |
| **Partner chromosome** | | chromosome 21 | | chromosome 22 | | chromosome 10 | | |
| **Most recent age at examination/sex** | | 17 months/M | | 17 years/M | | Patient 1- 19 years/M, Patient 2- 23 years/F, Patient 3- 23 years/M | | |
| **Short stature/growth retardation** | | + | | + | | -(1), +(2) | | |
| **Microcephaly** | | + | | + (relative microcephaly) | | + (1), brachycephaly (3) | | |
| **Eyes** | | NR | | up-slanting palpebral fissures, sparse lateral eyebrows | | up-slanting palpebral fissures (3), deep-set eyes (3), full upper eyelid (3) | | |
| **Ears** | | bilateral sensorineural hearing loss, low-set ears | | prominent | | NR(3) | | |
| **Nose** | | depressed nasal bridge, anteverted nares | | NR | | big nose (3) | | |
| **Mouth** | | long philtrum | | high arched palate, thick lips | | thick upper lip (3), short philtrum (3) | | |
| **Micrognathia** | | NR | | NR | | NR(3) | | |
| **Congenital heart defects** | | PVS, ASD | | - | | -(3) | | |
| **Upper airway malformation** | | - | | - | | NR(3) | | |
| **Skeletal anomalies** | | thoracic hemivertebrae, scoliosis, spina bifida, tethered spinal cord, hip dislocation | | - | | -(3) | | |
| **Extremities** | | - | | - | | brachydactyly (1) | | |
| **Urogenital anomalies** | | absence of right kidney, cryptorchidism, micropenis | | - | | micropenis and cryptorchidism (1) | | |
| **Mental retardation/development delay** | | + | | + (severe) | | +(2) severe, +(1) mild | | |
| **Hypertonia** | | abnormal muscle tone | | - | | -(1), NR (2) | | |
| **Hypotonia** | | + | | - | | -(1), NR(2) | | |
| **Seizures** | | + | | + | | +(2), -(1) | | |
| **Other** | | congenital diaphragmatic hernia, bilateral inguinal hernia, flattened occiput, short neck, weak primary reflexes | | self-injurious behaviour, mesenteric fibromatosis, atropic dermatitis, flattening of occiput, delayed puberty | | long face (3), insensitivity to pain (3), bitemporal narrowing (2), lung infection (1), hydrothorax (1), obesity (1), acanthosis nigricans (1), schizophrenia (1), nevi on the neck and face (3), abnormal EEG (1), abnormal brain imaging (3), empty sella (2), small pituitary (1), constant moving of extremities (1), autism spectrum disorder features (3), | | |
|  | | | | | | |  |  |

NR: Not Recorded; VSD: ventricular septal defect; PVS: pulmonary valve stenosis; PDA: patent ductus arteriosus; ASD: atrial septal defect.

- Means no abnormal findings; + Means abnormal findings
