# Supplementary material for: Molecular cytogenetic characterization of partial trisomy of the long arm of chromosome 11 in a patient with multiple congenital anomalies
Source: Mol Cytogenet. 2022 Apr 19;15:17. doi: 10.1186/s13039-022-00595-0 (PMC9019979; doi:10.1186/s13039-022-00595-0)
Supplement: Supplementary file 4 — Additional file 4. Table 4 Summaries of trisomy 11q cases from group 4. [file 13039_2022_595_MOESM4_ESM.docx]

| **TABLE 4** | *Burnside et al. (2009)* | *Forsythe et al. (1988)* | *Delobel et al. (1998)* |
| --- | --- | --- | --- |
| **Figure 3 reference number** | **16** | **17** | **18** |
| **Number of patients** | 1 (Patient 1) | 1 (Patient 2) | 1 |
| **Cytogenetics and molecular genetics findings** | 46,XY,rec(11)dup(11)(q23.3q24.2)ins(11)(q13.1q23.3q24.2)pat.ish dup(11)(q23.3q23.3)(*MLL*++).arrcgh11q23.3q24.2(RP11-447G4-> RP11-417F7) x 3. | 46,XX,rec(11),dup q,ins(11) (p14.2q23.3q24. 2) | 46,XX,dup(11) (q23.3q24?) |
| **Duplicated segment** | q23.3-q24.2 | q23.3–q24.2 | q23.3–24.? |
| **Partner chromosome** | none | none | none |
| **Most recent age at examination/sex** | 2 years/M | 11 years/F | 19 years/F |
| **Short stature/growth retardation** | - | - | <3rd centile |
| **Microcephaly** | + | <2nd centile | + |
| **Eyes** | - | strabismus, up-slanting palpebral fissures | strabismus, ptosis, prominent eyes |
| **Ears** | abnormal ears | NR | posteriorly angulated and anteverted, small but wide navicular crus |
| **Nose** | short nose | broad nose | beaked nose, large tip, oval nostrils |
| **Mouth** | thick lips | full lips | protruding upper lip, thick and everted lower lip, dental diastema, grinds teeth |
| **Micrognathia** | - | NR | NR |
| **Congenital heart defects** | NR | NR | - |
| **Upper airway malformation** | NR | NR | NR |
| **Skeletal anomalies** | - | scoliosis, hyperextensible joints, advanced bone age | scoliosis, short limbs |
| **Extremities** | clinodactyly | bilateral hindfoot deformities | cannot use hands, bilateral clinodactyly, small feet, crowded toes |
| **Urogenital anomalies** | NR | NR | NR |
| **Mental retardation/development delay** | + | + (mild to moderate) | + (severe) |
| **Hypertonia** | - | NR | pyramidal syndrome with hypertonicity and joint hyperlaxity |
| **Hypotonia** | - | NR | - (only after birth) |
| **Seizures** | - | NR | + |
| **Other** | NR | poor fine motor skills | triangular face, large forehead, low occipital hairline, hypertrichosis, intense vasomotor phenomena, trunk apraxia, ataxia, walks on tiptoes, stops breathing for 5-10 sec, hand washing/rubbing movements |

NR: Not Recorded; VSD: ventricular septal defect; PVS: pulmonary valve stenosis; PDA: patent ductus arteriosus; ASD: atrial septal defect.

- Means no abnormal findings; + Means abnormal findings
