# Supplementary material for: Molecular cytogenetic characterization of partial trisomy of the long arm of chromosome 11 in a patient with multiple congenital anomalies
Source: Mol Cytogenet. 2022 Apr 19;15:17. doi: 10.1186/s13039-022-00595-0 (PMC9019979; doi:10.1186/s13039-022-00595-0)
Supplement: Supplementary file 5 — Additional file 5. Table 5. Summaries of trisomy 11q cases from group 5. [file 13039_2022_595_MOESM5_ESM.docx]

| **TABLE 5** | *Legius et al. (1996)* | *Yelavarthi and Zunich (2004)* |
| --- | --- | --- |
| **Figure 3 reference number** | **19** | **20** |
| **Number of patients** | 1 | 1 |
| **Cytogenetics and molecular genetics findings** | 46,XX dir dup (11)(q13.3q14.2) | 46, XY dup(11)(q13.5q21).ish dup (11)(q13.5-q21)(wep11+)mat |
| **Duplicated segment** | q13.3–q14.2 | q13.5–q21 |
| **Partner chromosome** | Interstitial | Interstitial |
| **Most recent age at examination/sex** | 31 years/F | 6 years/M |
| **Short stature/growth retardation** | + (during childhood) | - |
| **Microcephaly** | - (10th centile) | - |
| **Eyes** | strabismus, right sided epicanthal fold | Downturned palpebral fissures, arched eyebrows |
| **Ears** | small ears, prominent antihelix | large ears, Darwinian tubercles, |
| **Nose** | NR | broad nasal root |
| **Mouth** | NR | Cupid's bow mouth, alveolar ridging with high arched and narrow palate, long philtrum |
| **Micrognathia** | retrognathia | NR |
| **Congenital heart defects** | VSD/PVS | NR |
| **Upper airway malformation** | NR | NR |
| **Skeletal anomalies** | pectus excavitum (severe), sacral dimple, normal dorsal kyphosis is absent | bithoracic narrowing, mid-thoracic spinal curvature, narrow shoulders |
| **Extremities** | joint hypermobility | broad fingers and thumbs, squared fingertips, bilateral transverse creases, missing left fourth and right third and fourth digital triradii |
| **Urogenital anomalies** | NR | NR |
| **Mental retardation/development delay** | + (mild to moderate) | + (mild) |
| **Hypertonia** | NR | NR |
| **Hypotonia** | neck and shoulder muscles hypotonic and hypotrophic with protruding scapulae | present at birth |
| **Seizures** | + | + |
| **Other** | thrombocytopenia, joint hypermobility, bleeding, menorrhagia, flat occiput | maternal drug and cigarette use suspected, inguinal hernia, aggressive behavior, sinus infections, apnea, tonsillectomy, myringotomy, adenoidectomy, trigonocephaly, sparse hair, frontal bossing, triangular facies, pointed chin, single café-au-lait spot on abdomen, attention problems, suspected abdominal migraines, recurrent headaches, |

NR: Not Recorded; VSD: ventricular septal defect; PVS: pulmonary valve stenosis; PDA: patent ductus arteriosus; ASD: atrial septal defect.

- Means no abnormal findings; + Means abnormal findings
